# Identification of COL4A2 as a Biomarker of Extracellular Matrix Remodeling and Vascular Scaffold in Choroid for Myopia

**DOI:** 10.1167/iovs.67.3.31

**Published:** 2026-03-13

**Authors:** Baizhou Chen, Yangming Xu, Songlin Xie, Xiang Zhou, Lin Wang, Chendong Yuan, Yao Ni, Zhaotian Zhang

**Affiliations:** 1 State Key Laboratory of Ophthalmology, Zhongshan Ophthalmic Center, Provincial Key Laboratory of Ophthalmology and Vision Science, Guangdong Provincial Clinical Research Center for Ocular Diseases, Sun Yat-Sen University, Guangdong, China; 2 Department of Pediatric Ophthalmology, Zhongshan Ophthalmic Center, Sun Yat-Sen University, Guangdong, China; 3 Medical Department, Guangzhou Medical University, Guangdong, China; 4 Department of Optometry, Zhongshan Ophthalmic Center, Sun Yat-Sen University, Guangdong, China

**Keywords:** extracellular matrix, choroid, collagen IV, form-deprivation myopia

## Abstract

**Purpose:**

To investigate the extracellular matrix (ECM) change in choroid and explore the key regulator of choroidal vascular scaffold in axial myopia.

**Methods:**

Myopia was induced in pigmented rabbits and guinea pigs using the form-deprivation approach. Quantitative label-free proteomics were performed using the samples of rabbits to investigate the differentially expressed proteins (DEPs) in myopic choroid. Gene Ontology (GO) and Kyoto Encyclopedia of Genes and Genomes (KEGG) enrichment was conducted to explore the potential biomarkers and signal pathways that form the scaffold and regulate the choroid morphology in myopia. Immunoblotting and immunofluorescence were applied to determine the expression and distribution of DEPs in choroid. Adeno-associated virus was used to knock down the expression of *COL4A2* to verify its biological function in refraction development and choroid morphology.

**Results:**

Long-term form deprivation significantly induced myopia shifts and decreased the choroidal thickness in pigmented rabbits. Proteomic analysis revealed that COL4A2 negatively regulates vasculature development. Masson’s trichome staining and immunofluorescence showed decreased choroidal thickness and lumen scaffold deformation with decreased collagen IV in form-deprivation myopia (FDM) choroid. Immunoblotting revealed that COL4A2 rather than COL4A1 contributes to the downregulation of collagen IV in FDM choroid. Dysfunction of COL4A2 hindered choroidal vascular scaffold formation, decreased guinea pig choroidal thickness, and promoted myopia shifts with refraction change and axial elongation.

**Conclusions:**

Type IV collagen is a key ECM in construction of the choroidal vascular lumen. COL4A2 is downregulated in FDM choroid, and suppression of COL4A2 impairs the choroid vascular scaffold and promotes myopia shift with decreased choroidal thickness.

Uncontrollable axial elongation in myopia jeopardizes visual acuity, carries blinding complications risks, and has emerged as a global public eye health problem.^1^^–^^3^ Choroid, a highly vascularized tissue, nourishes and transmits signals between retina and sclera and is considered to play a key role in axial myopia development.^4^^,^^5^ Choroid vascular dysfunction refers to reduced choroidal thickness and has been recognized as a sign of myopia pathogenesis.^6^ It has been confirmed that choroidal thickness and perfusion are strongly associated with refraction and axial length in myopia.^7^^,^^8^ Physical and pharmacological approaches to control myopia progression have also been shown to have significant influence in choroidal morphology.^9^^–^^12^ Nevertheless, the biomolecular characteristics and mechanisms of choroidal thickness change in myopia progression are still unclear. Hence, further exploration of the choroid dynamics and morphology in myopia is essential to reveal the mechanisms of myopia progression and identify myopia control targets.

The extracellular matrix (ECM) is a major constituent of the vascular lumen that maintains the structure of vessels and capillaries. Vascular ECM remodeling has been found to participate throughout the processes of circulation diseases, tumorigenesis, and choroidal neovascularization, for example.^13^^–^^15^ Collagen IV is one of the main types of ECM in vessel stroma that forms the basement membrane, and it is essential for endothelial cells and pericyte attachment and vasculogenesis. Deficiency of collagen IV may promote vasculopathy, such as glomerular nephropathy, intracerebral hemorrhage, and aortic aneurysm.^16^^–^^18^ Collagen IV genes are divided into two groups: (1) α1-like group, including COL4A1, COL4A3, and COL4A5; and (2) α2-like group, including COL4A2, COL4A4, and COL4A6.^19^ Three α chains self-assemble into a heterotrimeric promoter with two α1-like and one α2-like chain, and only three isoforms of promoters are recognized: α1α1α2 (IV), α3α4α5 (IV), and α5α5α6 (IV). Among those isoforms, α1α1α2(IV) is the predominant one widely located in the basement membrane of all tissues.^20^ Deficiency of the α1-like or α2-like chain interrupts collagen IV network formation, resulting in basement membrane abnormality and pathogenesis. Collagen IV is a crucial structural component of Bruch's membrane and choroidal basement membrane.^21^^,^^22^ However, the role of collagen IV in choroidal vasculogenesis and morphology change and its correlation with myopia progression have yet to be determined.

In this study, we investigated the expression profile of ECM based on form-deprivation myopia (FDM) in rabbits. When we compared the proteomic analysis of choroid samples between myopic and control groups, COL4A2 was identified as negatively regulating the vasculature of choroid. Additionally, we validated the hypothesis based on the FDM guinea pig model and further explored the impact of COL4A2 deficiency on choroid morphology and axial elongation. These findings provide new insights into myopic choroid vascular dysfunction and potential strategies to control myopia progression.

## Methods

### Animal Study

All animal experiments were approved by the Ethics Committee of Zhongshan Ophthalmic Center, Sun Yat-Sen University (Approval Nos. S2024021 and S2025003) and in concordance with the ARVO Statement for the Use of Animals in Ophthalmic and Vision Research.

Three-week-old pigmented rabbits and pigmented guinea pigs were purchased from a commercial supplier and reared in a 12-hour light (400–500 lux)/dark (0 lux) cycle with a room temperature of 25°C.

### FDM Induction

**Figure 1.**
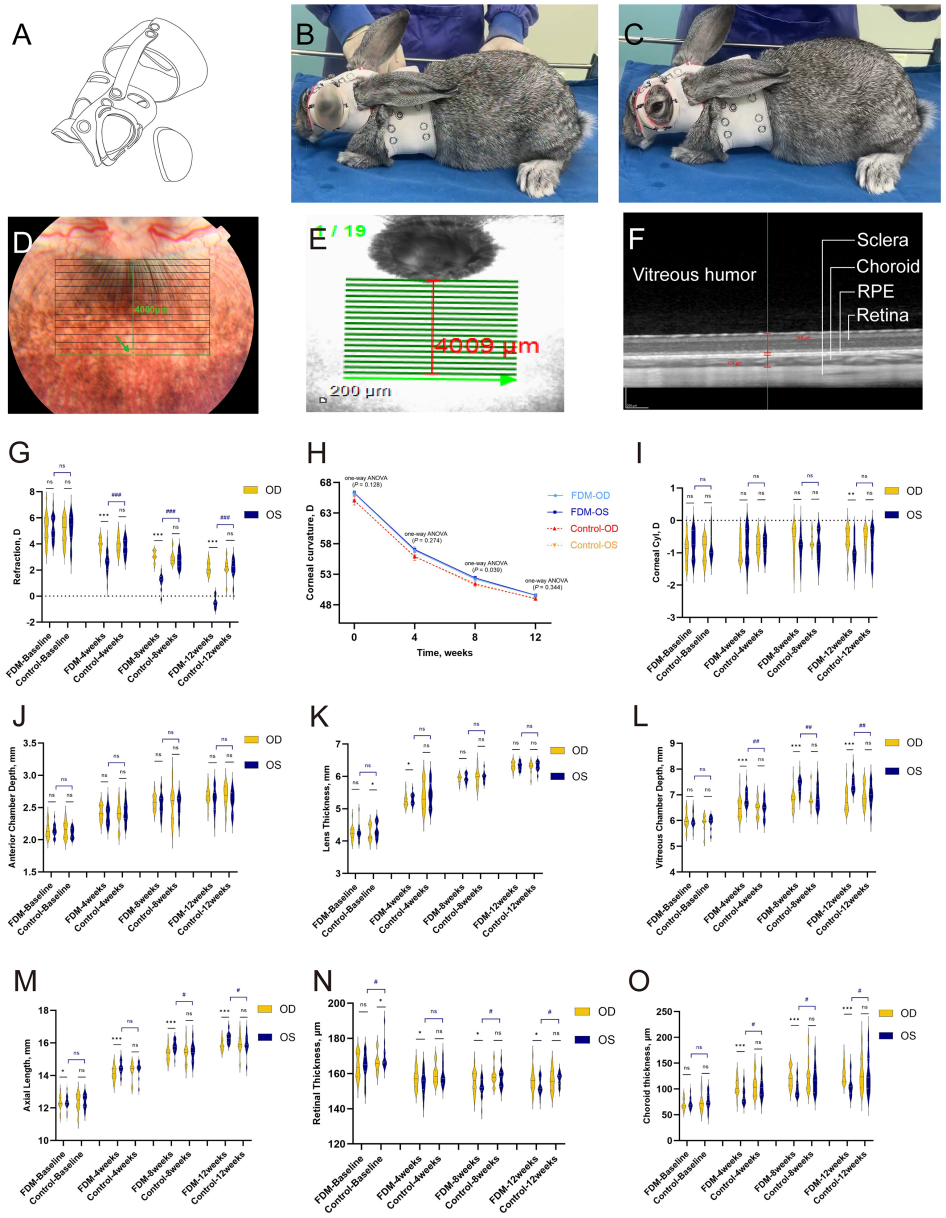
FDM induction in pigmented rabbits. (**A**–**C**) A 3D-printed diffuser was applied to pigmented rabbits to induce single-eye FDM. (**D**–**O**) The area of choroidal thickness analyzed was a horizontal zone located about 4000 µm tangentially to the lower edge of the ONH. Retinal thickness refers to the distance from the internal limiting membrane to the RPE layer, and choroidal thickness refers to the distance from the RPE layer to the choroid–sclera junction. Shown are 4-week time line changes in refraction, corneal curvature, corneal astigmatism, ACD, LT, VCD, AL, RT, and ChT from baseline to 12 weeks in the FDM group (*n* = 20) and control group (*n* = 11). *Yellow* violin plots show the measurement of right eyes, and *dark blue* plots represent the left eyes of the rabbits. The line chart shows the corneal curvature change across time; *light blue* represents the right eyes of the FDM group, *dark blue* represents the left eyes of the FDM group, *red* represents right eye of control group, and *orange* represents the left eyes of the control group. OD, oculus dexter; OS, oculus sinister. All data are presented as medians and quartiles. The *line in the middle* represents the median; the *upper dotted* line represents the third quartile, and the *lower dotted* *line* indicates the first quartile. Statistical comparisons included paired *t*-tests (*P *<* 0.05, **P *<* 0.01, ***P *<* 0.001) and unpaired *t*-tests (**^#^***P* *<* 0.05, **^###^***P* *<* 0.001; ns, not significant).

Rabbits were divided into FDM and control groups. The left eyes of rabbits in the FDM group were covered with a three-dimensional (3D)-printed removable opaque diffuser combined with a hood to induce FDM (Figs. 1A–C), and the right eyes were normally exposed. The diffusers were made of acrylonitrile butadiene styrene (ABS), and the hoods were constructed of terylene-spandex fabric. Diffusers were taken off once a day for cleaning and sterilizing with 75% ethanol to avoid infection (Supplementary Movie S1). The rabbit control group did not receive any cover for their eyes. The guinea pigs were also divided into FDM and control groups. In the FDM group, the left eye was covered with a latex balloon hood, and the right eye was exposed. The total cover duration was 2 weeks. The guinea pig control group did not receive any cover for their eyes.

### Ocular Parameters Measurement

Before measurement, all of the animals were treated with two applications of one drop of 1% cyclopentolate followed by one drop of 0.5% tropicamide/0.5% phenylephrine 5 minutes apart. Corneal curvature was measured using a keratometer, and refraction was measured using a streak retinoscope. Corneal curvature and refraction were measured twice by two experienced optometrists. Refraction was calculated using the average of vertical and horizontal meridian values and recorded as the spherical equivalent (SE). Animals with refraction less than 3 diopters (D), anisometropia greater than 1 D, or corneal cylindrical degree greater than 1.5 D were excluded. Animals were treated with 0.5% proparacaine before measurement of the ocular parameters. Anterior chamber depth (ACD), lens thickness (LT), vitreous chamber depth (VCD), and axial length (AL) were measured using A-scan ultrasonography. The sound velocities were set according to a previous study.^23^

The retina and choroid were scanned by spectral-domain optical coherence tomography (SD-OCT) with a wavelength of 870 nm. The scanning area in rabbits was defined as horizontal lines (30° × 20°, 8.6 × 5.7 mm) with 100 frames centered tangentially to the lower edge of the optic nerve head (ONH) (Fig. 1D). The scanning area in guinea pigs was defined as horizontal lines across the ONH (30° × 20°, 8.6 × 5.7 mm) with 100 frames. The enhanced depth imaging (EDI) mode was set to acquire accurate visualization of the choroid. This study analyzed scanning lines about 4000 µm away from the ONH rim in rabbits and scanning lines across the optic disc centrally in guinea pigs. Retinal thickness (RT) was defined as the distance between the inner limiting membrane and retinal pigment epithelium (RPE), and choroidal thickness (ChT) was defined as the distance from the RPE layer to the choroid–sclera junction. To avoid the interference of circadian rhythms, OCT scanning was performed between 3:00 PM and 8:00 PM.

### Proteomic Analysis

Rabbit choroid samples of the FDM eyes and contralateral eyes were isolated at 12 weeks for label-free quantitative proteomics analysis. Eight samples (four pairs of eyes) were prepared for proteomics analysis. Proteins were extracted and digested by trypsin/Lys-C combination enzymes to prepare peptide samples. All peptide mixture samples were analyzed on an Orbitrap Exploris 480 mass spectrometer (Thermo Fisher Scientific, Waltham, MA, USA). The raw data generated were processed using Proteome Discoverer 2.4. Results were compared with a mass spectrometry database for *Oryctolagus cuniculus* (Uniprot version, https://www.uniprot.org).

The protein abundance of each sample was analyzed. Differentially expressed protein (DEP) screening and enrichment analysis were conducted in R 4.3.1 (R Foundation for Statistical Computing, Vienna, Austria). Missing values were imputed using the K-nearest neighbor algorithm in the R impute package. Data were transformed logarithmically into an expression matrix, and the false discovery rate (FDR) was limited to <1% to calculate the DEPs. Upregulated proteins were defined as those with a log_2_ fold change (log_2_FC) > 0.585, and downregulated proteins were defined as those with a log_2_FC < −0.585. Gene Ontology (GO) enrichment was conducted according to the g:Profiler tool. Kyoto Encyclopedia of Genes and Genomes (KEGG) enrichment was conducted using the Bioconductor package clusterProfiler 4.0.5.

### Hematoxylin and Eosin Staining

Eyes were enucleated and fixed in a ferrous ammonium sulfate solution for 24 hours. Tissues were embedded in paraffin. After deparaffinization and dehydration, 5-µm vertical sections were taken and stained using a hematoxylin and eosin (H&E) staining kit. Histology images were acquired using a scanning confocal microscope under white light.

### Masson's Trichrome Staining

Masson's trichrome staining was conducted, and scans were acquired using a scanning confocal microscope. In the Masson's trichrome images, the blue-stained area indicated mature collagen deposition. Collagen volume fraction (CVF) was defined as the percentage of blue-stained area divided by the total targeted area.

### Immunofluorescence

#### Paraffin Immunofluorescence

Sections were dewaxed and underwent antigen retrieval. After blocking with goat serum, sections were incubated with primary antibodies at 4°C overnight. Before confocal microscope scanning, Alexa Flour 488/594–conjugated secondary bodies and 4′,6-diamidino-2-phenylindole (DAPI) staining were applied and the sections were incubated for 2 hours. Images were captured by confocal microscopy.

#### Frozen Immunofluorescence

Eyes were enucleated and preserved at −80°C. Ocular tissues were embedded in a mixture of optimal cutting temperature (O.C.T.) compound and sucrose mixture for 2 hours. They were then transferred into O.C.T. compound for 4 hours, and sections were taken at −20°C. After blocking with goat serum, the sections were incubated with primary antibodies at 4°C overnight. Before the confocal microscope scanning, the samples were incubated for 2 hours with Alexa Flour 647–conjugated secondary bodies and DAPI staining for 2 hours. Images were captured by the confocal microscope.

### Capillary Immunoblot

Choroid samples were collected and reserved at −80°C. Protein extraction was performed using radioimmunoprecipitation assay (RIPA) buffer and zirconia beads in the grinder. After centrifugation, sediments were discarded, and the supernatants were collected to calculate the concentration using the bicinchoninic acid assay. Protein expression was determined using the automated ProteinSimple Wes western blot system (ProteinSimple, Inc., San Jose, CA, USA). Wes separation capillary cartridges for 12 to 230 kDa and 66 to 440 kDa were applied for target protein detection. Primary antibodies against the targets and secondary antibodies are listed in the Supplementary Materials. Signals were detected with horseradish peroxidase (HRP)-conjugated secondary antibodies. Results were analyzed using Compass for SW 6.3.0 (Bio-Techne, Minneapolis, MN, USA).

### AAV Vector Design and Suprachoroidal Injection

The self-complementary adeno-associated virus (scAAV) carrying short hairpin RNA (AAV-shCOL4A2) and negative control (AAV-NC) were obtained from PackGene Biotech (Guangzhou, China). Titers of the two viruses were about 1 × 10^13^ virus particles per milliliter. Sequences of shRNA and the scAAV plasmid vector are provided in the Supplementary Fig. S1.

Guinea pigs were anesthetized with isoflurane. A conjunctival incision was made using microscissors to expose the sclera. Then, 10 µL of AAV was injected suprachoroidally through the sclera using a microsyringe. The pseudo-surgery (sham) group received only the conjunctival incision and needle insertion. After injection, tobramycin–dexamethasone ointment was applied to prevent infection, and the animals were placed on a thermostatic mat for resuscitation (Supplementary Movie S2).

### Statistical Analysis

For analysis of the data for the FDM and control groups in pigmented rabbits and guinea pigs, parametric paired *t*-tests were applied to compare the differences in measurements between the left and right eyes. Unpaired *t*-tests were applied to compare the differences between the left eyes (covered eyes) in the FDM group and left eyes in the control group, including refraction, corneal astigmatism, ACD, LT, VCD, AL, RT, and ChT. Normality and variance tests were conducted before the *t*-test. One-way ANOVA analysis was applied to compare the corneal curvature of the pigmented rabbits, followed by Bonferroni correction. For data of the suprachoroidal injection group, multiple comparisons using least significant difference (LSD; homogeneous variance) or Dunnett’s *t*-test (heterogeneous variance) were applied to analyze the differences among the AAV injection, sham, control, and FDM groups, depending on the variance test results. All statistical analyses and data graphic presentations were conducted using the SPSS Statistics 25.0 (IBM, Chicago, IL, USA) and Prism 9 (GraphPad, Boston, MA, USA). *P* < 0.05 was considered statistically significant.

All of the equipment and materials used are listed in the Supplementary Materials.

## Results

### Refraction, Ocular Parameters, RT, and ChT Change in Long-Term Induced Form-Deprivation Pigmented Rabbits

To evaluate the efficacy and stability of the diffuser to induce FDM, refraction, cornea curvature, corneal astigmatism, and AL were measured. At baseline, no significant difference was found in refraction of the covered eyes (left eyes) and exposed eyes (right eyes) in the FDM group (mean difference = 0 D; 95% confidence interval [CI], −0.17 to 0.17; *P* > 0.05). No significant differences were found in cornea curvature, corneal astigmatism, ACD, LT, VCD, RT, or ChT between the left and right eyes in the FDM group. AL measurements showed a slight distinction between the covered eyes and exposed eyes at baseline (mean difference = 0.03 mm; 95% CI, 0.01–0.05; *P* = 0.028). No significant difference in refraction was found between left eyes and right eyes in the control group (mean difference = 0.09 D; 95% CI, −0.17 to 0.35; *P* > 0.05). Also, no significant differences were observed in corneal curvature, corneal astigmatism, ACD, VCD, AL, or ChT. Slight differences were found between the left and right eyes in LT (mean difference = 0.08 mm; 95% CI, 0.02–0.13; *P* = 0.021) and RT (mean difference = 3.42 µm; 95% CI, 0.76–3.42; *P* = 0.03) (Figs. 1G–O)

After 12 weeks, the covered eyes in the FDM group developed myopia (−0.45 ± 0.41 D), and the contralateral (uncovered) eyes were hyperopic (2.15 ± 0.43 D), resulting in a significant relative myopic shift in the covered eyes (mean difference = −2.6 D; 95% CI, −2.80 to −2.40; *P* < 0.001). Covered eyes showed longer VCD (mean difference = 0.64 mm; 95% CI, 0.55–0.73; *P* < 0.001) and AL (mean difference = 0.49 mm; 95% CI, 0.39–0.58; *P* < 0.001) than exposed eyes. Also, thinner RT (mean difference = −2.48 µm; 95% CI, −4.56 to −0.41; *P* = 0.03) and ChT (mean difference = −21.62 µm; 95% CI, −27.53 to −15.70; *P* < 0.001) were observed in covered eyes compared to the contralateral eyes. Eyes in the control group showed no difference in refraction, ocular parameters, RT, or ChT. A negligible difference was found in corneal astigmatism in FDM rabbits (−1.0 ± 0.41 D vs. −0.6 ± 0.4 D in covered and exposed eyes, respectively), but corneal curvature remained similar. These results indicated that the self-designed diffuser could cover the FDM-induced eye stably without impairing corneal refraction (Figs. 1G–O).

### Proteomic-Based ECM Expression Profile Revealed DownRegulation of COL4A2 in the Choroid of FDM

**Figure 2.**
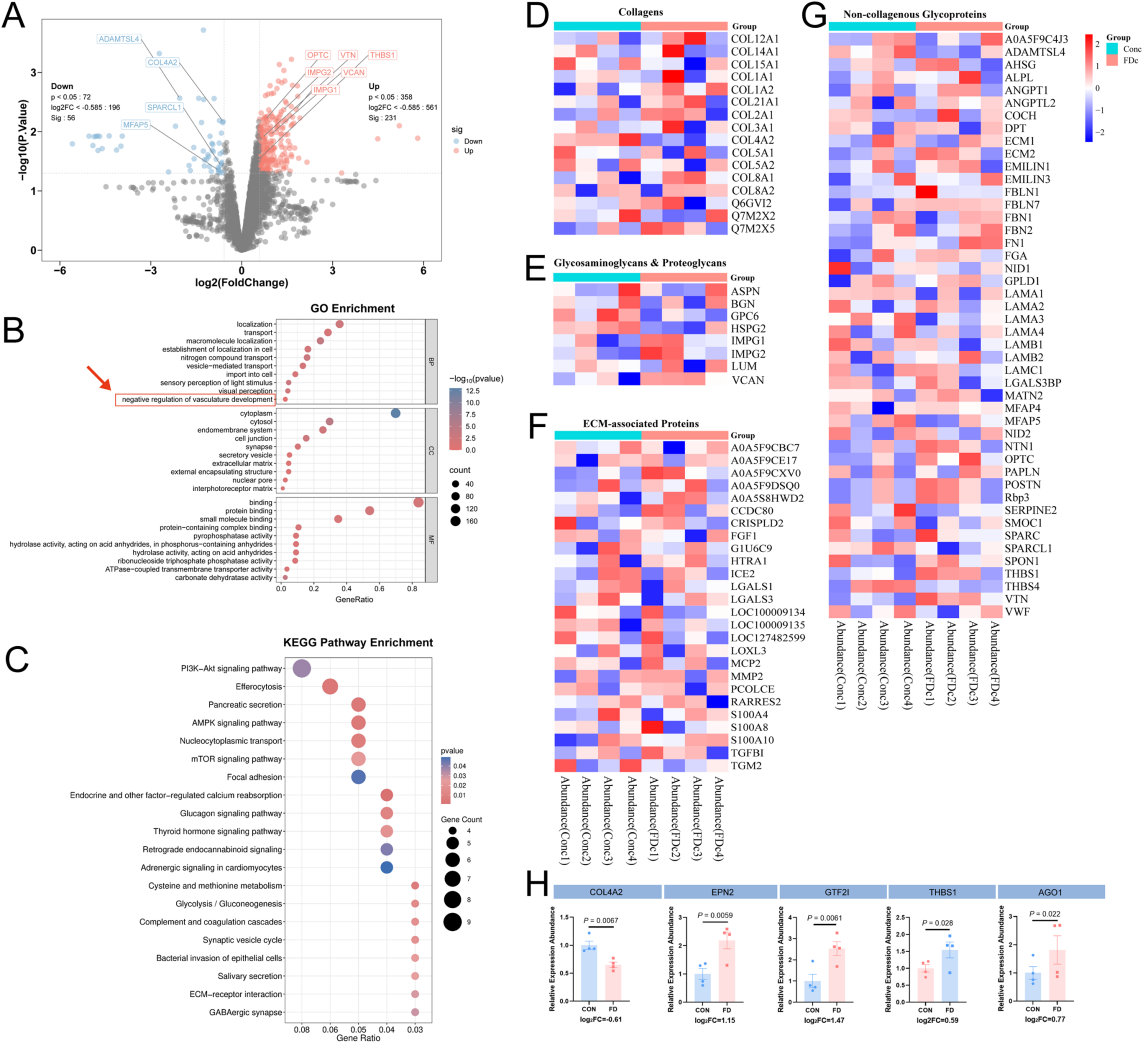
Label-free proteome analysis of the choroid of the paired eyes from form-deprivation pigmented rabbits (*n* = 4). (**A**) The volcano plot shows DEPs and ECM proteins. (**B**, **C**) GO enrichment and KEGG enrichment analyses showed the biological processes, cellular components, molecular functions, and signal pathways related to the DEPs. (**D**–**G**) ECM heatplot shows the abundance profile of different types of ECM when compared between FDM eyes and the contralateral eyes. (**H**) DEPs probably contributed to the negative regulation of vasculature development; biological processes are listed to show the fold changes between FDM and contralateral eyes.

Because the ECM is essential to vasculature development, we hypothesized that ECM remodeling would be correlated with choroid changes in myopia. To investigate ECM changes in the choroid of FDM, we conducted quantitative label-free proteomic analysis using the samples obtained at 12 weeks from the induced FDM pigmented rabbits (*n* = 4). A total of 4561 proteins and peptides were identified. Volcano plots revealed 231 upregulated and 56 downregulated proteins (Fig. 2A).

GO enrichment analysis was conducted to elucidate the biological processes, cellular components, and molecular functions of DEPs. Interestingly, negative regulation of vasculature development, a biological process component, was enriched and probably correlated with choroidal thickness reduction in myopia. KEGG pathway enrichment analysis showed that DEPs were enriched in phosphoinositide 3-kinase (PI3K)/Akt signaling, AMP-activated protein kinase (AMPK) signaling, and mammalian target of rapamycin (mTOR) signaling pathways, the top three enriched pathways. The ECM–receptor interaction pathway revealed that ECM may be involved in myopic choroidal change (Figs. 2B, 2C).

To determine the predominant ECM associated with myopic choroidal changes, the ECM was classified into four types: (1) collagens, (2) glycosaminoglycans and proteoglycans, (3) non-collagenous glycoproteins, and (4) ECM-associated proteins, based on a previous study.^24^ In total, five upregulated and four downregulated ECM proteins were identified (Figs. 2A, 2D–G). DEPs enriched for negative regulation of vasculature development included COL4A2, EPN2, GTF2I, THBS1, and AGO1 (Fig. 2H), suggesting that they have a role in regulating choroid morphology during myopia progression. Sankey plots showed the correlation between DEPs and the enriched pathways (Supplementary Fig. S2).

**Figure 3.**
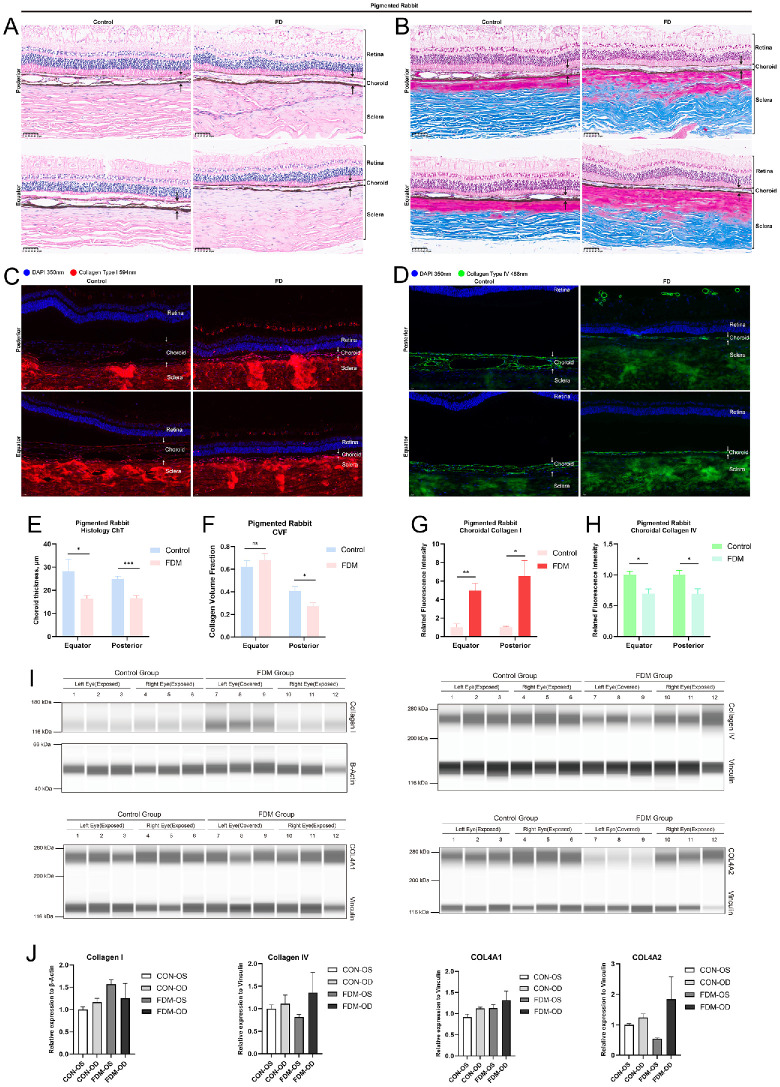
Histology and protein expression differences between the FDM and control groups in rabbit choroid. (**A**) H&E staining shows the histology differences between the FDM and control eyes of rabbits in the equator and posterior area of eyeball. (**B**) Masson's trichrome staining shows the collagen distribution in rabbit eyes; the *blue-stained* *area* represents collagen deposition. (**C**, **D**) Immunofluorescence shows the distribution of collagen IV and collagen I in the fundus of rabbits eyes; the *dark green bar* represents the fluorescence intensity of the collagen in the control group, and the *light green bar* represents the fluorescence intensity in the FDM group. (**E**, **F**) Choroidal thickness was measured as the distance from the RPE to the choroid–sclera junction; the collagen volume fraction was calculated as the ratio of *blue-stained* *area* and total area of choroid; the *blue bar* represents the control group, and the *red bar* represents the FDM group. (**G**, **H**) The *dark red* and *pink bars* represent the fluorescence intensity of collagen I in the control and FDM groups, respectively. (**I**, **J**) Immunoblotting showed the relative expression of collagen IV, collagen I, COL4A1, and COL4A2 in rabbit choroid. All data are presented as mean ± SEM. Statistical analysis included unpaired *t*-tests (*P *<* 0.05, **P *<* 0.01, ***P *<* 0.001; ns, not significant).

Network-forming collagen IV is the main constituent of the basement membrane of choriocapillaris, as noted previously.^25^ Deficiency of collagen IV α-chain leads to vasculopathy and causes disease, indicating that COL4A2 would be key in regulating choroidal morphology in myopia. Also, collagen type I overexpression has been reported in myopic choroid.^26^ To compare morphology changes and collagen distributions in choroid, H&E and Masson's trichrome staining were applied. The control group showed thicker and more lumen-like structure in the choroid compared with the FDM group (Figs. 3A, 3E). Posterior areas showed higher CVFs in the control group than in the FDM group (Figs. 3B, 3F). To confirm the distribution of two types of collagens in choroid, immunofluorescence was applied to visualize their location and expression in the rabbit samples. Results revealed that collagen I was distributed in choroid stroma and that collagen IV was a component of the vascular lumen wall. Higher fluorescence intensity of collagen IV was observed in the control group than in the FDM group (Figs. 3C, 3D, 3H). Also, the fluorescence intensity of collagen I in choroid was weaker than that in the FDM group (Fig. 3G).

To determine the expression levels of collagen IV and collagen I, choroid samples of rabbits were collected and analyzed by the Wes western blot system. Collagen IV was downregulated and collagen I was upregulated in the FDM eyes compared to the contralateral and control group eyes. Considering the heterotrimeric structure of collagen IV, COL4A1- and COL4A2-specific antibodies were applied to further explore their expression. Western blot revealed significant downregulation of COL4A2 in FDM eyes but COL4A1 did not show any difference (Figs. 3I, 3J).

### DownRegulation of COL4A2 Was Also Observed in FDM Guinea Pigs

**Figure 4.**
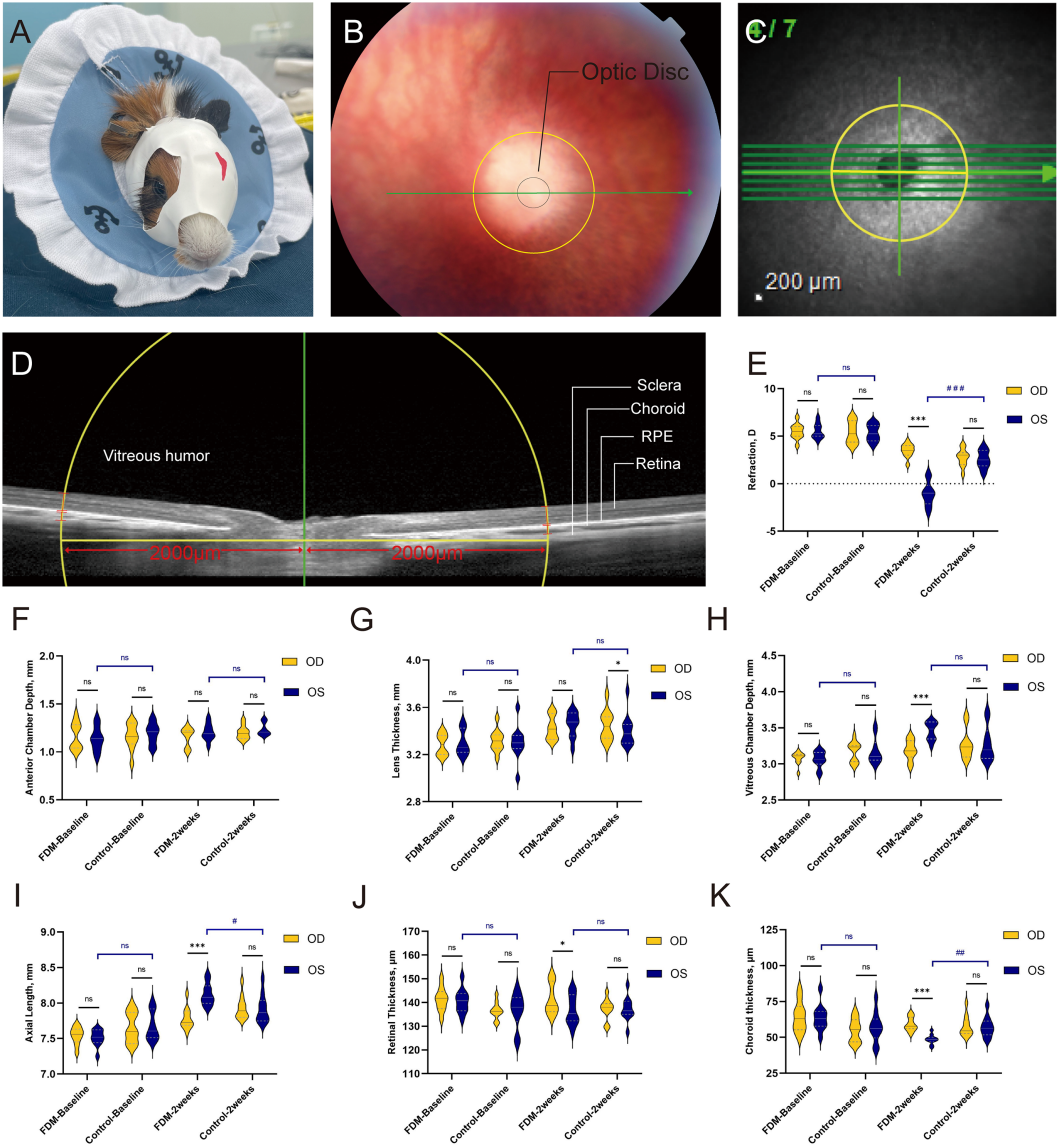
FDM induction in guinea pigs. (**A**) A latex balloon hood was applied to pigmented guinea pigs to induce single-eye FDM. (**B**–**D**) Choroidal thickness area analyzed was selected as the horizontal line about 2000 µm away from the nasal and temporal edge of the ONH. Retinal thickness refers to the distance from the internal limiting membrane to the RPE layer, and choroidal thickness refers to the distance from the RPE layer to the choroid–sclera junction. (**E**–**K**) Shown are time line changes in refraction, AL, ACD, LT, VCD, RT, and ChT from baseline to 2 weeks in the FDM group (*n* = 10) and the control group (*n* = 10). *Yellow* violin plots show the measurement of right eyes, and *dark blue* plots represent the left eyes of the guinea pigs. All data are presented as medians and quartiles. The *line in the middle* represents the median; the *upper dotted* *line* represents the third quartile, and the lower dot line shows the first quartile. Statistical comparisons included paired *t*-tests (*P *<* 0.05, **P *<* 0.01, ***P *<* 0.001; ns, not significant) and unpaired *t*-tests (^#^*P* *<* 0.05, ^##^*P* *<* 0.01, ^###^*P* *<* 0.001; ns, not significant).

Guinea pigs are widely used in myopia research. To investigate whether FDM guinea pigs would show changes similar to those observed in the rabbits, pigmented guinea pigs were randomly divided into FDM and control groups. The FDM group wore a cover for 2 weeks on the left eye but the right eye was exposed (Fig. 4A). Refraction (mean difference = 0.05 D; 95% CI, −0.22 to 0.32; *P* = 0.726) and AL (mean difference = −0.03 mm; 95% CI, −0.06 to 0.00; *P* = 0.063) showed no significant differences between covered and exposed eyes. No significant differences were found for baseline choroidal thickness (mean difference = −0.55 µm; 95% CI, −3.38 to 2.28; *P* = 0.713) or other parameters (Figs. 4E–K).

After 2 weeks of FDM induction, refraction between the covered and exposed eyes showed a significant difference (mean difference = −4.58 D; 95% CI, −5.30 to −3.85; *P* < 0.001). Elongated VCD (mean difference = 0.26 mm; 95% CI, 0.19–0.34; *P* < 0.001) and AL (mean difference = 0.34 mm; 95% CI, 0.29–0.40; *P* < 0.001) were found in the covered eyes compared to exposed eyes. No statistical significance was found in ACD or LT. ChT values in covered eyes were reduced significantly compared to exposed eyes (mean difference = −10.6 µm; 95% CI, −12.78 to −8.42; *P* < 0.001) (Figs. 4E–K).

**Figure 5.**
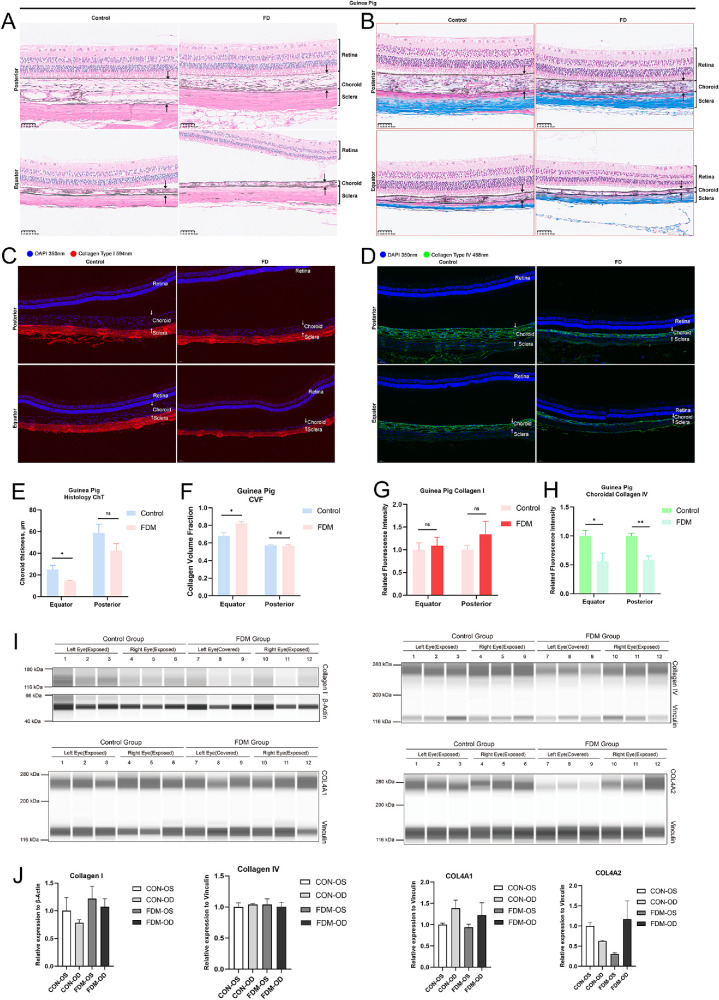
Histology and protein expression differences between the FDM and control groups in guinea pig choroid. (**A**) H&E staining shows the histology differences between the FDM and control eyes of guinea pigs in the equator and posterior area of eyeball. (**B**) Masson's trichrome staining shows the collagen distribution in guinea pig eyes; the *blue-stained* area represents collagen deposition. (**C**, **D**) Immunofluorescence shows the distribution of collagen IV and collagen I in the fundus of guinea pig eyes; the *dark green bar* represents the fluorescence intensity of the collagen in control group and light green bar represents the fluorescence intensity in the FDM group. (**E**, **F**) Choroidal thickness was measured as the distance from the RPE to the choroid–sclera junction; the collagen volume fraction was calculated as the ratio of *blue-stained* *area* and total area of choroid; the *blue bar* represents the control group, and the *red bar* represents the FDM group. (**G**, **H**) The *dark red* and *pink bars* represents the fluorescence intensity of collagen I in the control and FDM groups, respectively. (**I**, **J**) Immunoblotting showed the relative expression of collagen IV, collagen I, COL4A1, and COL4A2 in guinea pig choroid. All data are presented in mean ± SEM. Statistical analysis included unpaired *t*-tests (*P *<* 0.05, **P *<* 0.01, ***P *<* 0.001; ns, not significant).

Histology showed thinner choroid and fewer lumen structures in FDM eyes than in the control eyes, and Masson's staining revealed higher CVFs in the equatorial area of the FDM group (Fig. 5A, 5B, 5E–F). Immunofluorescence showed similar distributions of collagen IV in choroid. Higher fluorescence intensity was observed in the control group than in the FDM group (Fig. 5D, 5H). Western blot showed significant downregulation of COL4A2 in the FDM group, but the expression of COL4A1 showed no difference. Also, no differences in fluorescence intensity and expression levels of collagen I were observed in the FDM and control groups (Figs. 5C, 5G, 5I).

### Deficiency of COL4A2 Promotes Myopic Choroid Vascular Dysfunction Shift and Axial Elongation

**Figure 6.**
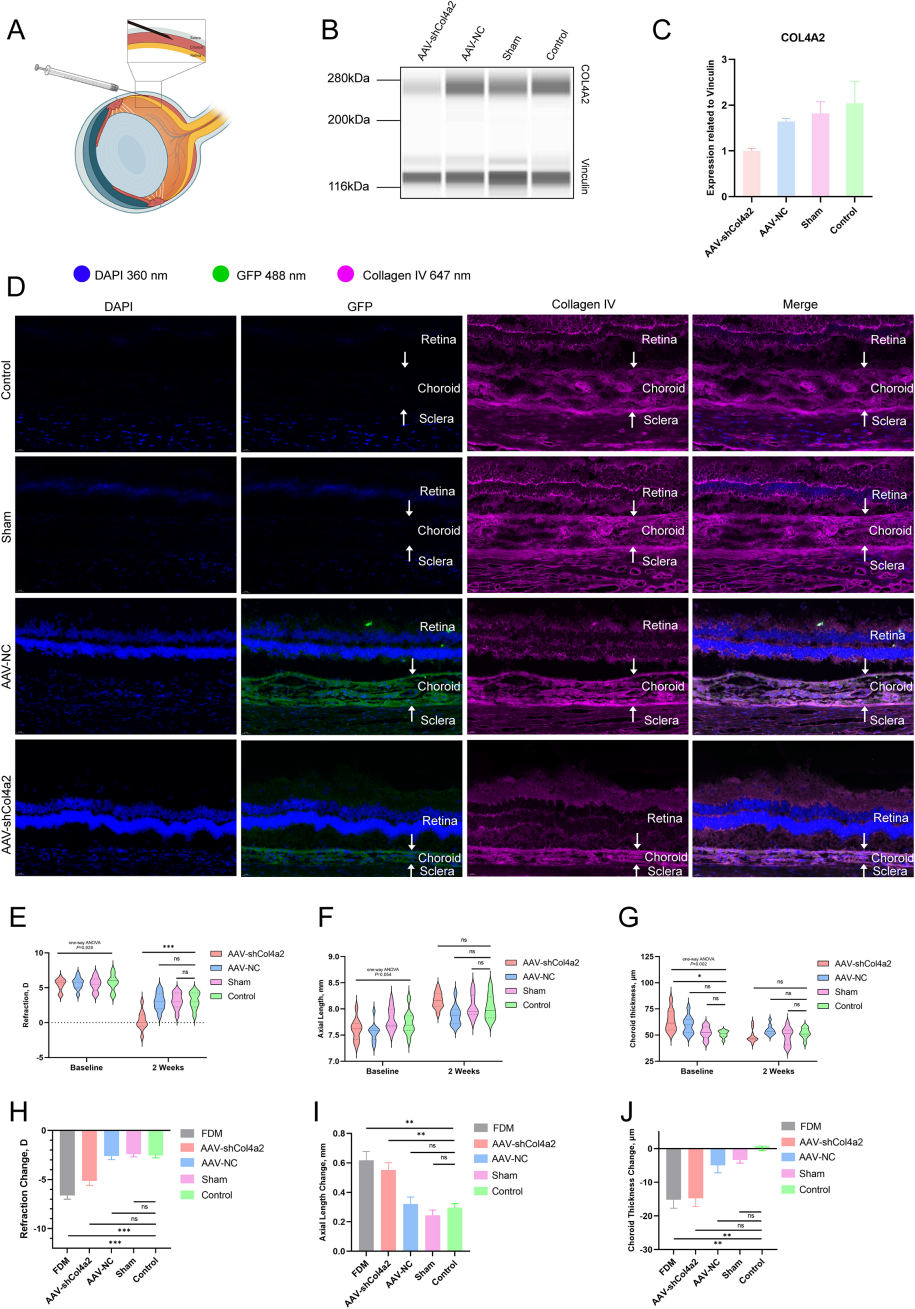
Suppression of COL4A2 and ocular parameters in guinea pigs with suprachoroidal injection. (**A**) Suprachoroidal injection was performed using a Hamilton 34-gauge microinjection needle through the sclera posterior 1 mm to the limbus. (**B**, **C**) Immunoblotting showed that the expression of collagen IV α2 chains in the choroid of guinea pigs was suppressed after AAV-shCOL4A2 delivery. (**D**) Immunofluorescence revealed the distribution of GFP expression in the choroid of guinea pigs. Green fluorescence at 488 nm showed that the AAV-NC (*n* = 10) and AAV-shCOL4A2 (*n* = 10) were correctly injected into the choroid. Eyes in the sham group (*n* = 9) underwent the surgical process without AAV injection, and eyes in the control group (*n* = 9) did not receive any intervention. Magenta fluorescence at 647 nm showed the distribution of collagen IV in frozen sections. (**E**–**G**) Violin plots show the measurements of refraction, AL, choroidal thickness, and their time change at baseline and at 2 weeks. The data are presented as medians (*line*) and for the first and third quartiles (*dotted* *lines*). Calculated time change data from baseline to 2 weeks are shown in bar charts, and data are presented as mean ± SEM. Statistical comparisons were conducted using one-way ANOVA and multiple comparisons with the LSD test when variance was homogeneous; otherwise, Dunnett’s *t*-test was used. *P *<* 0.05, **P *<* 0.01, ***P *<* 0.001; ns, not significant.

The experiments above indicated that collagen IV is crucial for choroidal lumen construction and choroidal scaffold maintenance. To determine the effect of a deficiency of COL4A2 on choroidal thickness and myopia progression, we designed shRNA to knock down the expression of COL4A2 in the choroid of guinea pigs via suprachoroidal injection (Fig. 6A). After 2 weeks, 488-nm signals showed that AAV had been integrated into the choroidal cells with green fluorescent protein (GFP) expression (Fig. 6D). Western blot showed suppression of COL4A2 in the choroid of AAV-shRNA–injected eyes (Figs. 6B, 6C).

After 2 weeks, refraction of the control group decreased by 2.56 D (95% CI, −3.03 to −2.08). VCD increased by 0.15 mm (95% CI, 0.04–0.26), and AL increased by 0.30 mm (95% CI, 0.24–0.35). Refraction of the AAV-shCOL4A2–injected eyes significantly decreased by 5.15 D (95% CI, 4.28–6.02). VCD and AL significantly increased by 0.3 mm (95% CI, 0.20–0.40) and 0.55 mm (95% CI, 0.46–0.65), respectively, compared to the control group. However, compared to the control group, no significant change was found in refraction, VCD, or AL of AAV-NC–injected eyes and the sham group. OCT scanning also revealed significantly decreased ChT from baseline to the 2-week endpoint in eyes injected with AAV-shCOL4A2 (−14.8 µm; 95% CI, −19.52 to −10.08) compared to the AAV-NC group (−5 µm; 95% CI, −9.33 to −0.67), sham group (−3.39 µm; 95% CI, −5.21 to −1.56), and control group (0.83 µm; 95% CI, −1.98 to 3.65). 2 weeks of FDM induction shows myopia shift with refraction changes (−6.63 D; 95% CI, −7.36 to −5.89) and axial elongation (0.62 mm; 95% CI, 0.490.74). 2 weeks of COL4A2 suppression showed similar effects on guinea pigs, both refraction change (−5.15 D; 95% CI, −6.02 to −4.28) and AL changes (0.55 mm; 95% CI, 0.460.65) support the evidence of a myopia shift. These results indicated that a deficiency of COL4A2 potentially decreases choroidal thickness and promotes axial elongation (Figs. 6E, 6F, 6J).

## Discussion

In this study, we induced FDM in pigmented rabbits and guinea pigs to explore the role of choroidal ECM in myopia progression. Consistent with observations in other clinical studies, choroidal thickness in form-deprivation eyes was decreased compared to the contralateral eyes and the control group. Proteomic analysis findings revealed the potential function of COL4A2 in regulating choroid morphology. Inhibiting the expression of COL4A2 decreased choroidal thickness in both OCT scanning and histology, along with changes in refraction and axial length in accordance with myopia progression.

Several species were used to induce myopia for research. Although quick and effective induction of myopia has been reported in mice, guinea pigs, and chicks, their poor vision and eye scale restricts their application.^27^ Monkeys have the highest similarity with humans in anatomy; however, most form-deprivation–treated eyes in rhesus monkeys require a long-term induction (81 days or more) to reach negative lens power.^28^^,^^29^ Due to the high costs and poor compliance, it is difficult to conduct myopia research on monkeys. Rabbits serve as a common animal model for myopia research, as they have larger-scale eyes. Lower costs and good compliance facilitate the use of rabbits in research. Rabbits have been widely used in pharmacological explorations.^30^^,^^31^ Previous studies have reported that the refraction of pigmented rabbits decreased from 4.89 ± 0.51 D to 1.83 ± 0.7 D after an 8-week form-deprivation induction, and the refraction of albino rabbits decreased from 3.06 ± 0.4 D to 0.03 ± 0.47 D after a 6-week induction.^23^^,^^32^ In our study, refraction of the covered eyes decreased from 5.21 ± 0.89 D to −0.45 ± 0.41 D, and AL increased from 12.36 ± 0.37 mm to 16.31 ± 0.32 mm after 12 weeks of form-deprivation induction. Choroidal thickness of the rabbits was found to grow with time; ChT in form-deprivation–induced eyes showed slower growth compared to the contralateral eyes and the control group (at 12 weeks, change across time of form-deprivation eyes: 34.9 ± 12.43 µm; contralateral eyes: 58.12 ± 10.84 µm; control group eyes: 51.06 ± 25.52 µm). These results indicate that the 3D-printed diffuser effectively induced FDM in rabbits. Also, this diffuser served as a tool for topical eyedrop administration at a high frequency during induction.

Recent studies on the choroid dynamics, including choroidal thickness, vascular lumen ratio, and blood perfusion, have verified the relationship between the choroid and myopia progression. A lower choroidal vascular index (defined as ratio of luminal area and total choroid area) is accompanied by axial elongation, indicating the progression of pathological myopia.^8^ Myopia control approaches, such as atropine solutions, defocus lenses, multifocal contacts lenses, and low-level red light, have been shown to effectively increase choroidal thickness.^9^^–^^12^ Animal experiments have found that ciliary artery transection reduces choroidal thickness and promotes myopia progression; also, increases in choroidal blood perfusion inhibit axial elongation and myopia shifts.^33^^,^^34^

ECM is the primary structural constituent of vascular stroma, and ECM remodeling is essential in perfusion homeostasis regulation and vasculopathy. Collagen IV is the crucial component and the most abundant collagen in basement membrane; it serves as the skeleton for vasculogenesis during eye development and has a role in supporting lymphatic vessel development.^35^ It is the main component of Bruch's membrane and choroidal stroma.^22^^,^^36^ Collagen IV provides attachment sites for endothelial cells and pericytes to form the lumen. Gene deletion of collagen IV results in defects in capillary formation, reduced capillary density and vessel integrity, whereas the development of large vessels is not affected.^37^ COL4A1 and COL4A2 mutants cause broken basement membranes, leading to endothelium dysfunction and intracerebral hemorrhage.^16^ Downregulation of COL4A1 in endothelial cells affects capillary formation. Inhibition of collagen IV synthesis by *cis*-hydroxyproline–blocked tube formation and exogenous addition of collagen IV promotes tube formation.^38^ Our study found that choroidal COL4A2 is deficient in myopia eyes, which probably impairs α1α1α2 trimeric promoter assembly and damages the matrix necessary to form lumen and the vasculature of endothelial cells and pericytes.

Although the synthesis regulation and how choroid collagen IV interacts with choroidal cells in myopia remain undetermined, many studies have revealed the expression-regulating cascade and binding sites of collagen IV in other research fields. In mesangial cells, high-glucose–induced collagen IV expression through mTOR/Akt signaling could be attenuated by forkhead box P1 (FOXP1) protein.^39^ High-glucose levels also upregulated collagen IV and activated Akt signaling in RPE cells, but the PI3K inhibitor LY294002 abolished this process.^40^ Collagen IV acts as a ligand to bind with cell-surface receptors and regulates biological processes. In currently available research, integrin (ITG) and discoidin domain receptor (DDR) have been the most discussed. The non-collagenous domain of collagen IV binds to integrin and affects a number of signaling processes in endothelial cells and pericytes.^41^ DDR1 is reported to combine with COL4A3 and exacerbate renal fibrosis in mice.^42^ In our study, KEGG enrichment showed that several signaling pathways were enriched with a high gene ratio, including PI3K/Akt signaling, efferocytosis, and AMPK signaling. Sankey plots illustrated a connection between COL4A2 and the enriched pathways; however, whether these pathways relate to COL4A2 regulation and how these pathways work in regulating choroidal thickness and myopia progression require further investigation (Supplementary Figs. S2, S3).

There are some limitations in this research. The current study conducted a long-term induction of form deprivation in rabbits and analyzed measurements taken at baseline and endpoints, but the intermediate time points were not further analyzed. Although two species were included in our study, the lack of human samples and experimental and clinical evidence of a relationship between COL4A2 mutation and myopia weakens the broader impact. COL4A2 has been identified as playing a major role in choroid vascular morphology in myopia; however, there is a lack of information regarding how it influences choroidal scaffold formation and how the changed choroid regulates axial elongation. Also, the long coding sequences of COL4A2 make it difficult to conduct rescue experiments using AAV vector, and it is still unclear whether COL4A2 precedes choroidal thinning or is a result of it. Single-cell and CRISPR activation techniques could be used to further explore the choroid morphology regulation mechanism of COL4A2 in myopia.

In conclusion, we found that long-term form deprivation induced axial myopia shifts in pigmented rabbits. FDM choroid had less lumen structure than the control group. Collagen IV is a major component of the lumen wall of choriocapillaris, and downregulation of COL4A2 was observed in the choroid of form-deprivation eyes. Inhibiting the expression of COL4A2 reduces choroidal thickness and promotes axial myopia shifts, indicating that COL4A2, as a component of the basement membrane, supports lumen formation and could serve as a biomarker of vascular ECM remodeling in the choroid.

## Supplementary Material

Supplement 1

Supplement 2

Supplement 3

Supplement 4

## Supplementary Material

**Supplementary Movie S1.** Removable opaque diffuser to induce FDM in rabbit.

**Supplementary Movie S2.** Suprachoroidal injection of guinea pig.
