# Supplementary material for: Identification of COL4A2 as a Biomarker of Extracellular Matrix Remodeling and Vascular Scaffold in Choroid for Myopia
Source: Invest Ophthalmol Vis Sci. 2026 Mar 13;67(3):31. doi: 10.1167/iovs.67.3.31 (PMC13020118; doi:10.1167/iovs.67.3.31)
Supplement: Supplement 1 [file iovs-67-3-31_s001.docx]

Supplementary Materials-Figures


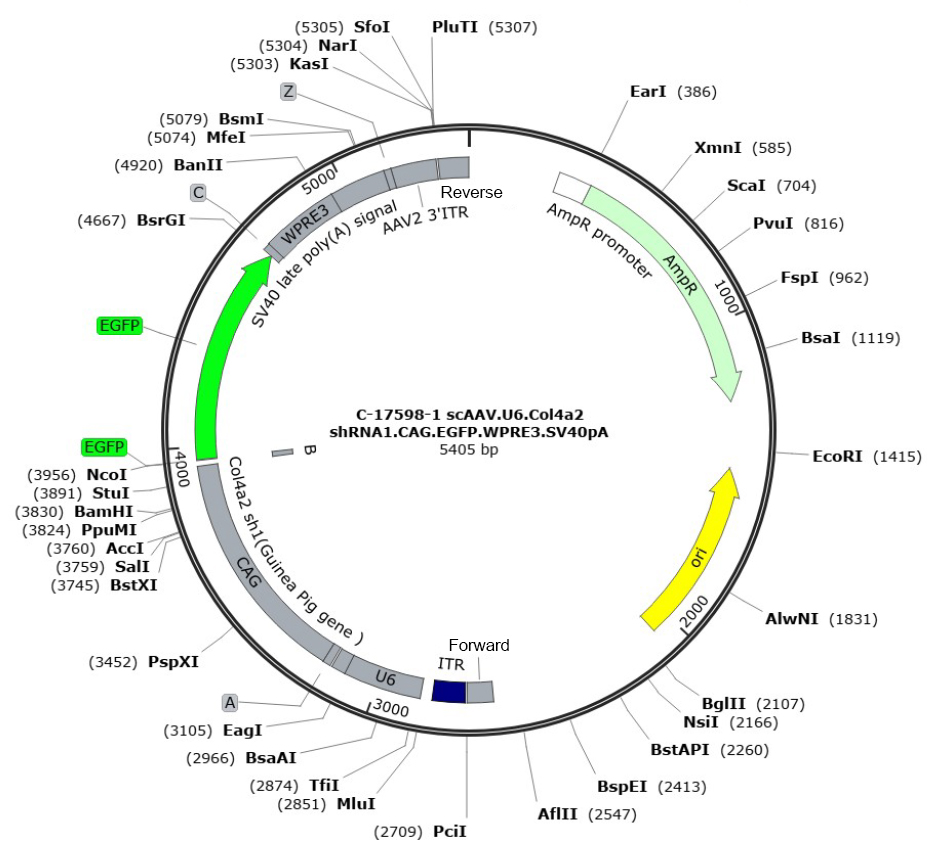


Figure S1. Plasmid Vector of shCol4a2


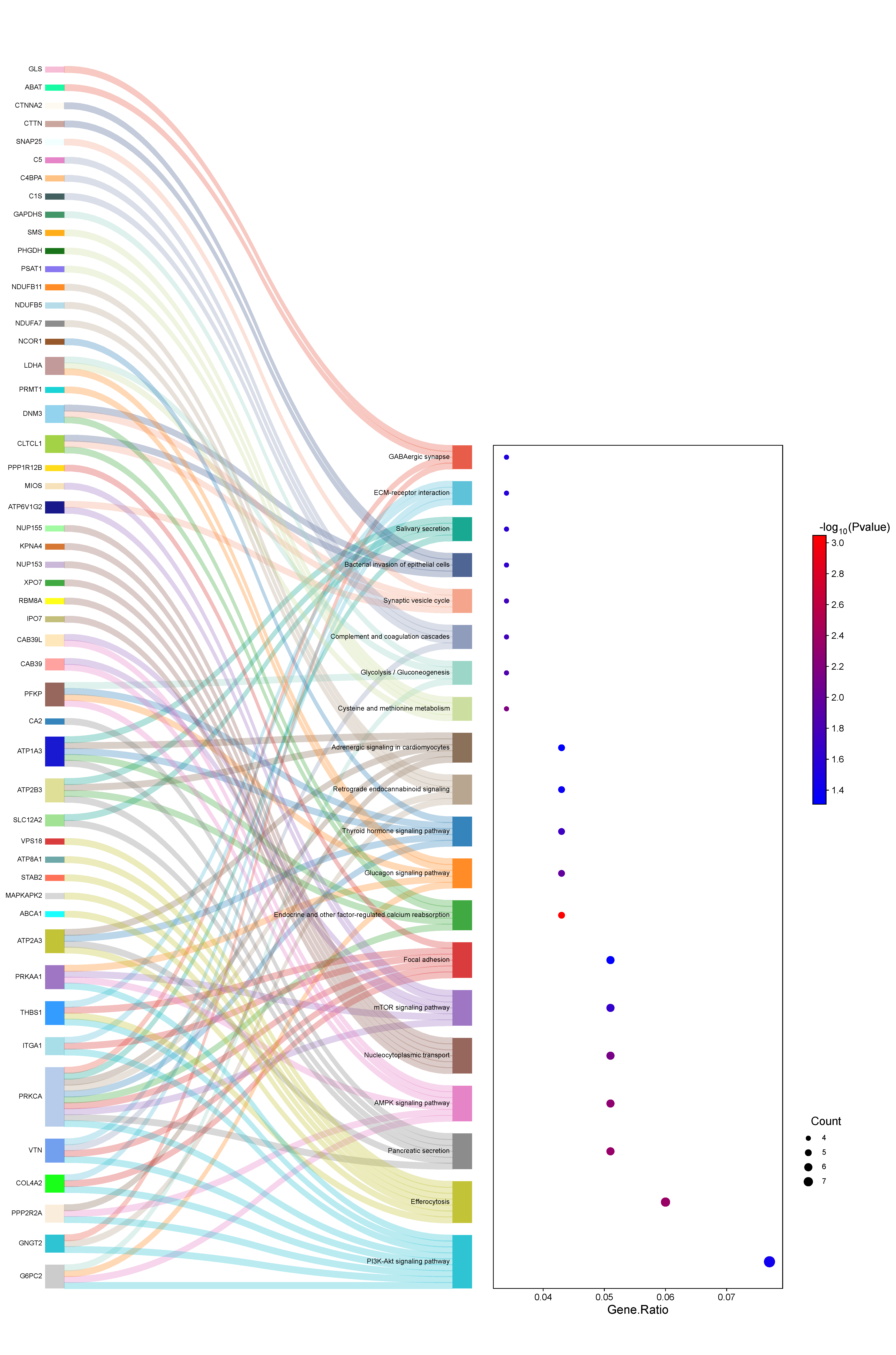


Figure S2. Sankey Plot of DEPs


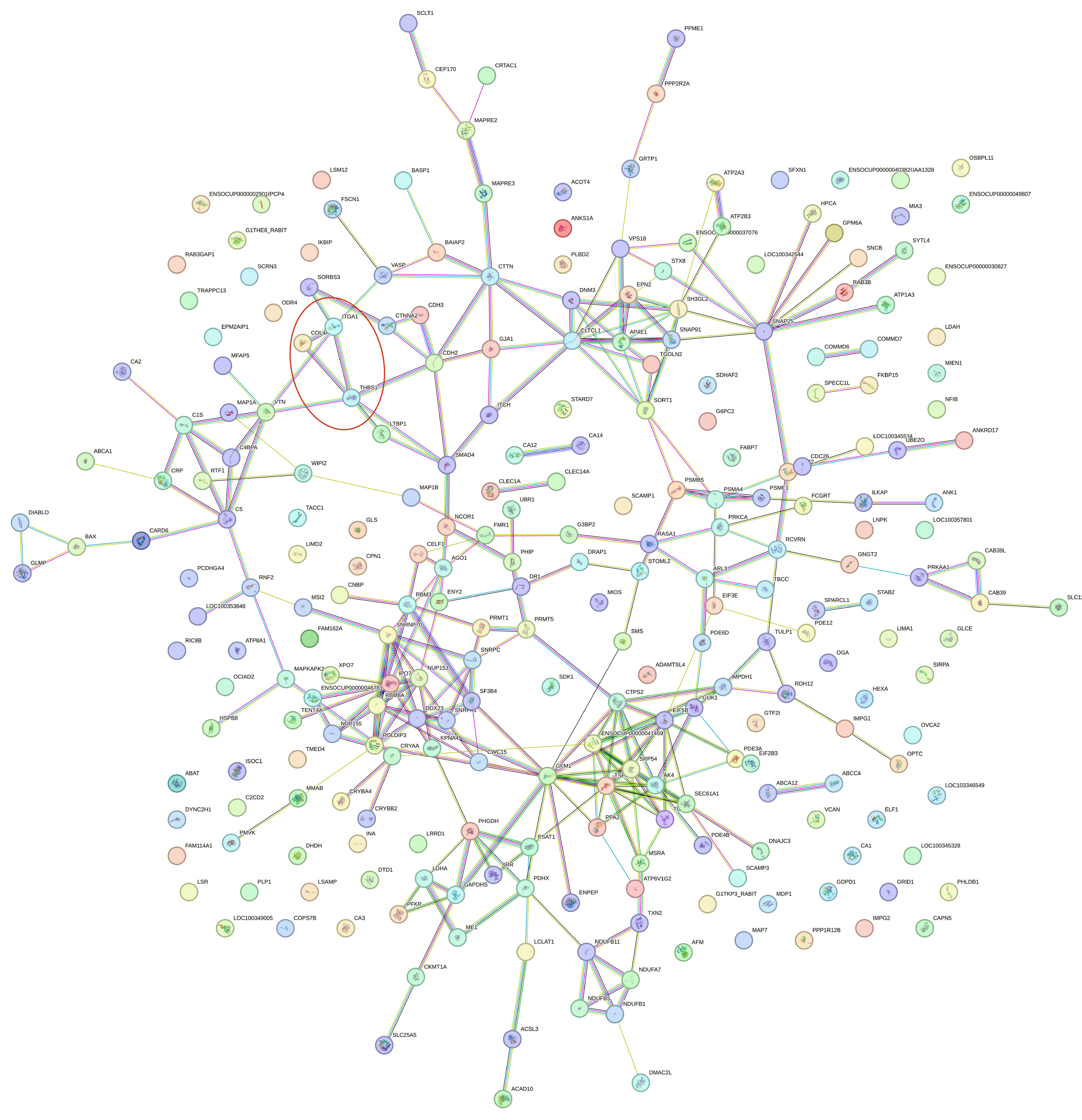


Figure S3. Protein-protein interaction network. Red ellipse shows the interaction between COL4A2, ITGA1
