# Supplementary material for: Identification of COL4A2 as a Biomarker of Extracellular Matrix Remodeling and Vascular Scaffold in Choroid for Myopia
Source: Invest Ophthalmol Vis Sci. 2026 Mar 13;67(3):31. doi: 10.1167/iovs.67.3.31 (PMC13020118; doi:10.1167/iovs.67.3.31)
Supplement: Supplement 2 [file iovs-67-3-31_s002.docx]

Supplementary Materials-Tables

Table S1. Rabbits refraction and corneal curvature across time

| **Group** | **Time** | **RE, D** | |  | **CCR, D** | |  | **Corneal Cyl, D** | |  |
| --- | --- | --- | --- | --- | --- | --- | --- | --- | --- | --- |
|  |  | **OD(Expose)** | **OS(Cover)** | ***P-*value** | **OD(Expose)** | **OS(Cover)** | ***P-*value** | **OD(Expose)** | **OS(Cover)** | ***P-*value** |
| **FDM**  **(n=20)** | baseline | 5.21±1.01 | 5.21±0.89 | 1.0 | 66.21±1.61 | 66.29±1.68 | 0.343 | -0.91±0.38 | -0.73±0.43 | 0.065 |
|  | 4 weeks | 3.99±0.5 | 2.63±0.8 | <0.001 | 56.9±1.76 | 57.04±1.94 | 0.134 | -0.91±0.36 | -0.78±0.52 | 0.248 |
|  | 8 weeks | 3.08±0.4 | 1.11±0.59 | <0.001 | 52.21±1.17 | 52.4±1.32 | 0.044 | -0.65±0.49 | -0.85±0.45 | 0.159 |
|  | 12 weeks | 2.15±0.43 | -0.45±0.41 | <0.001 | 49.53±0.98 | 49.58±1.05 | 0.451 | -0.6±0.4 | -1±0.41 | 0.007 |
|  | Change  (baseline-12 weeks) | -3.06±1.11 | -5.66±1.06 | <0.001 | -16.69±1.44 | -16.71±1.53 | 0.868 | 0.31±0.53 | -0.28±0.56 | <0.001 |
|  |  |  |  |  |  |  |  |  |  |  |
|  |  | **OD(Expose)** | **OS(Expose)** |  | **OD(Expose)** | **OS(Expose)** |  | **OD(Expose)** | **OS(Expose)** |  |
| **Control**  **(n=11)** | baseline | 4.89±1.03 | 4.98±1.27 | 0.506 | 65±2.28 | 65±2.52 | 1.0 | -0.91±0.36 | -0.82±0.4 | 0.58 |
|  | 4 weeks | 3.82±0.73 | 3.75±0.67 | 0.518 | 55.89±2.22 | 55.95±2.26 | 0.653 | -0.75±0.30 | -0.77±0.33 | 0.892 |
|  | 8 weeks | 2.82±0.6 | 2.75±0.62 | 0.539 | 51.43±1.28 | 51.23±1.26 | 0.095 | -0.64±0.23 | -0.82±0.43 | 0.195 |
|  | 12 weeks | 2.18±0.68 | 2.14±0.68 | 0.617 | 49±0.97 | 49.14±1 | 0.465 | -0.57±0.33 | -0.75±0.5 | 0.152 |
|  | Change  (baseline-12 weeks) | -2.7±0.84 | -2.84±0.88 | 0.294 | -16.05±1.73 | -15.86±2.04 | 0.441 | 0.5±0.83 | 0.07±0.59 | 0.097 |

All the comparisons were conducted in paired t-test. Normality and variance testing were conducted before the parametric test.

Table S2. Rabbits ocular biometry parameters across time

| **Group** | **Time** | **ACD, mm** | |  | **LT, mm** | |  | **VCD, mm** | |  | **AL, mm** | |  |
| --- | --- | --- | --- | --- | --- | --- | --- | --- | --- | --- | --- | --- | --- |
|  |  | **OD(Expose)** | **OS(Cover)** | ***P*-value** | **OD(Expose)** | **OS(Cover)** | ***P*-value** | **OD(Expose)** | **OS(Cover)** | ***P*-value** | **OD(Expose)** | **OS(Cover)** | ***P*-value** |
| **FDM**  **(n=20)** | baseline | 2.15±0.12 | 2.14±0.09 | 0.811 | 4.25±0.19 | 4.25±0.22 | 0.851 | 5.99±0.28 | 6.01±0.27 | 0.337 | 12.33±0.38 | 12.36±0.37 | 0.028 |
|  | 4 weeks | 2.42±0.12 | 2.38±0.14 | 0.279 | 5.24±0.17 | 5.3±0.15 | 0.019 | 6.49±0.37 | 6.86±0.33 | <0.001 | 14.13±0.39 | 14.49±0.37 | <0.001 |
|  | 8 weeks | 2.58±0.11 | 2.55±0.16 | 0.394 | 5.93±0.12 | 5.95±0.13 | 0.326 | 6.79±0.35 | 7.36±0.31 | <0.001 | 15.31±0.38 | 15.78±0.35 | <0.001 |
|  | 12 weeks | 2.68±0.12 | 2.63±0.18 | 0.165 | 6.33±0.13 | 6.32±0.12 | 0.401 | 6.76±0.41 | 7.40±0.28 | <0.001 | 15.85±0.29 | 16.33±0.30 | <0.001 |
|  | Change  (baseline-12 weeks) | 0.54±0.18 | 0.49±0.15 | 0.324 | 2.08±0.19 | 2.07±0.22 | 0.65 | 0.78±0.35 | 1.39±0.31 | <0.001 | 3.52±0.32 | 3.97±0.33 | <0.001 |
|  |  |  |  |  |  |  |  |  |  |  |  |  |  |
|  |  | **OD(Expose)** | **OS(Expose)** |  | **OD(Expose)** | **OS(Expose)** |  | **OD(Expose)** | **OS(Expose)** |  | **OD(Expose)** | **OS(Expose)** |  |
| **Control**  **(n=11)** | baseline | 2.16±0.13 | 2.11±0.08 | 0.084 | 4.27±0.23 | 4.35±0.24 | 0.021 | 5.95±0.3 | 5.95±0.24 | 0.984 | 12.38±0.50 | 12.41±0.41 | 0.434 |
|  | 4 weeks | 2.39±0.18 | 2.43±0.16 | 0.351 | 5.41±0.43 | 5.44±0.38 | 0.189 | 6.47±0.33 | 6.48±0.36 | 0.812 | 14.28±0.54 | 14.36±0.5 | 0.104 |
|  | 8 weeks | 2.55±0.25 | 2.53±0.16 | 0.761 | 5.96±0.26 | 5.99±0.15 | 0.507 | 6.82±0.35 | 6.92±0.45 | 0.245 | 15.34±0.56 | 15.44±0.55 | 0.071 |
|  | 12 weeks | 2.68±0.17 | 2.60±0.17 | 0.096 | 6.29±0.17 | 6.32±0.14 | 0.433 | 6.96±0.41 | 6.98±0.46 | 0.698 | 15.94±0.58 | 15.91±0.62 | 0.118 |
|  | Change  (baseline-12 weeks) | 0.52±0.21 | 0.49±0.21 | 0.438 | 2.02±0.25 | 1.97±0.27 | 0.17 | 1.01±0.39 | 1.03±0.38 | 0.788 | 3.56±0.63 | 3.49±0.67 | 0.221 |

All the comparisons were conducted in paired t-test. Normality and variance testing were conducted before the parametric test.

Table S3. Rabbits retinal thickness and choroid thickness across time

| **Group** | **Time** | **RT, μm** | |  | **ChT, μm** | |  |
| --- | --- | --- | --- | --- | --- | --- | --- |
|  |  | **OD(Expose)** | **OS(Cover)** | ***P*-value** | **OD(Expose)** | **OS(Cover)** | ***P*-value** |
| **FDM**  **(n=20)** | baseline baseline | 164.15±6.74 | 164.3±7.24 | 0.917 | 70.67±10.32 | 72.27±10.72 | 0.129 |
|  | 4 weeks | 157.13±5.42 | 155.02±6.33 | 0.047 | 105.05±16.31 | 88.18±16.49 | <0.001 |
|  | 8 weeks | 155.38±5.71 | 152.42±5.15 | 0.024 | 121.15±17.96 | 101.23±16.35 | <0.001 |
|  | 12 weeks | 155.52±5.82 | 153.03±4.58 | 0.03 | 128.78±17.52 | 107.17±19.52 | <0.001 |
|  | Change  (baseline-12weeks) | -8.63±6.59 | -11.27±6.04 | 0.128 | 58.12±10.84 | 34.9±12.43 | <0.001 |
|  |  |  |  |  |  |  |  |
|  |  | **OD(Expose)** | **OS(Expose)** |  | **OD(Expose)** | **OS(Expose)** |  |
| **Control**  **(n=11)** | baseline | 166.67±5.49 | 170.09±7.83 | 0.03 | 74.3±16.55 | 76.48±16.08 | 0.282 |
|  | 4 weeks | 159.76±5.3 | 158.85±5.71 | 0.493 | 106.85±20.91 | 102.97±20.03 | 0.13 |
|  | 8 weeks | 158.21±4.98 | 157.45±5.14 | 0.584 | 123.45±29.91 | 118.82±26.2 | 0.116 |
|  | 12 weeks | 155.93±5.77 | 156.91±3.45 | 0.628 | 129.39±31.64 | 127.55±35.2 | 0.256 |
|  | Change  (baseline-12 weeks) | -10.73±5.86 | -13.18±7.03 | 0.333 | 55.09±23.67 | 51.06±25.52 | 0.051 |

All the comparisons were conducted in paired t-test. Normality and variance testing were conducted before the parametric test.

| **Group** | **Measurements** | **Baseline** | |  | **2 Weeks** | |  |
| --- | --- | --- | --- | --- | --- | --- | --- |
|  |  | **OD(Expose)** | **OS(Cover)** | ***P-*value** | **OD(Expose)** | **OS(Cover)** | ***P-*value** |
| **FDM**  **(n=10)** | RE, D | 5.5±0.82 | 5.55±0.8 | 0.726 | 3.5±0.71 | -1.08±1.28 | <0.001 |
|  | ACD, mm | 1.17±0.14 | 1.14±0.13 | 0.547 | 1.16±0.1 | 1.21±0.09 | 0.262 |
|  | LT, mm | 3.28±0.09 | 3.3±0.11 | 0.593 | 3.43±0.1 | 3.46±0.11 | 0.388 |
|  | VCD, mm | 3.08±0.09 | 3.06±0.12 | 0.456 | 3.19±0.14 | 3.46±0.13 | <0.001 |
|  | AL, mm | 7.54±0.12 | 7.51±0.12 | 0.063 | 7.78±0.15 | 8.13±0.16 | <0.001 |
|  | RT, μm | 141.35±5 | 140.65±5.09 | 0.702 | 141.45±6.53 | 136.95±6.26 | 0.022 |
|  | ChT, μm | 64.35±10.55 | 63.8±9.19 | 0.713 | 59.2±4.79 | 48.6±3.41 | <0.001 |
|  |  |  |  |  |  |  |  |
|  |  | **OD(Expose)** | **OS(Expose)** |  | **OD(Expose)** | **OS(Expose)** |  |
| **Control1**  **(n=10)** | RE, D | 5.4±1.17 | 5.35±0.88 | 0.758 | 2.75±0.92 | 2.55±0.96 | 0.104 |
|  | ACD, mm | 1.16±0.14 | 1.19±0.11 | 0.24 | 1.2±0.07 | 1.24±0.07 | 0.09 |
|  | LT, mm | 3.32±0.09 | 3.3±0.15 | 0.536 | 3.45±0.13 | 3.4±0.14 | 0.017 |
|  | VCD, mm | 3.17±0.14 | 3.18±0.18 | 0.844 | 3.28±0.21 | 3.28±0.24 | 0.966 |
|  | AL, mm | 7.65±0.21 | 7.68±0.21 | 0.305 | 7.94±0.17 | 7.92±0.2 | 0.618 |
|  | RT, μm | 136.55±3.65 | 136.65±7.46 | 0.962 | 137.2±4.57 | 137.25±5.18 | 0.977 |
|  | ChT, μm | 54.8±8.27 | 57.2±10.75 | 0.242 | 57.85±7.33 | 57.3±7.23 | 0.527 |

Table S4. Guinea Pig refraction, ocular biometry, retinal thickness and choroid thickness across time

All the comparisons were conducted in paired t-test. Normality and variance testing were conducted before the parametric test.

Table S5. Guinea Pig with suprachoroidal injection refraction, ocular biometry, retinal thickness and choroid thickness across time

| **Timepoint** | **Measurements** | **Group** | | | |  |
| --- | --- | --- | --- | --- | --- | --- |
|  |  | **AAV-shCol4a2**  **(n=10)** | **AAV-NC**  **(n=10)** | **Sham**  **(n=9)** | **Control2**  **(n=9)** | **one-way ANOVA** |
| **Baseline** | RE, D | 5.45±0.86 | 5.65±0.85 | 5.53±0.99 | 5.72±1.18 | 0.929 |
|  | ACD, mm | 1.1±0.06 | 1.06±0.06 | 1.14±0.07 | 1.13±0.1 | 0.11 |
|  | LT, mm | 3.34±0.13 | 3.34±0.16 | 3.38±0.1 | 3.38±0.12 | 0.839 |
|  | VCD, mm | 3.16±0.18 | 3.15±0.18 | 3.28±0.24 | 3.22±0.19 | 0.492 |
|  | AL, mm | 7.61±0.21 | 7.56±0.18 | 7.8±0.22 | 7.74±0.23 | 0.054 |
|  | RT, μm | 142.3±7.2 | 143.8±6.38 | 137.78±4.27 | 137±5.18 | 0.079 |
|  | ChT, μm | 63.8±10.21^#^ | 59.55±8.31 | 52.28±6.06 | 51.06±3.3 | 0.002 |
|  |  |  |  |  |  |  |
| **2 weeks** | RE, D | 0.3±1.35^#^ | 3.05±1.23 | 3.11±1.17 | 3.17±1.2 | <0.001 |
|  | ACD, mm | 1.2±0.06^#^ | 1.1±0.06 | 1.15±0.05 | 1.14±0.09 | 0.013 |
|  | LT, mm | 3.49±0.12 | 3.51±0.1 | 3.54±0.08 | 3.52±0.09 | 0.749 |
|  | VCD, mm | 3.46±0.14 | 3.26±0.17 | 3.34±0.23 | 3.37±0.22 | 0.16 |
|  | AL, mm | 8.16±0.15 | 7.88±0.19 | 8.05±0.22 | 8.03±0.23 | 0.031 |
|  | RT, μm | 135.2±6.41 | 137.8±6.1 | 131±6.61 | 137.39±4.89 | 0.133 |
|  | ChT, μm | 49±6.21 | 54.55±4.71 | 48.89±8.1 | 51.89±4.98 | 0.148 |

All data presented in Mean±SD, unpaired t-test applied. Normality and variance testing were conducted before the parametric test. Significant difference: one-way ANOVA multiple comparison *P* <0.05, # AAV-shCol4a2 vs Control2; † AAV-NC vs Control2; § Sham vs Control2;

Table S6. Guinea Pig refraction, ocular biometry, retinal and choroid thickness time change.

|  | **Measurements** | **Group** | | | | |
| --- | --- | --- | --- | --- | --- | --- |
|  |  | **FDM**  **(n=10)** | **AAV-shCol4a2**  **(n=10)** | **AAV-NC**  **(n=10)** | **Sham**  **(n=9)** | **Control2**  **(n=9)** |
| **Change**  **(baseline-2weeks)** | RE, D | -6.63±1.19^*^ | -5.15±1.4^#^ | -2.6±1.15 | -2.42±0.85 | -2.56±0.73 |
|  | ACD, mm | 0.07±0.18 | 0.11±0.08 | 0.04±0.09 | 0.02±0.05 | 0.01±0.15 |
|  | LT, mm | 0.15±0.14 | 0.15±0.06 | 0.17±0.1 | 0.13±0.11 | 0.16±0.11 |
|  | VCD, mm | 0.4±0.09^*^ | 0.4±0.16^#^ | 0.11±0.11 | 0.06±0.16 | 0.15±0.17 |
|  | AL, mm | 0.62±0.19^*^ | 0.55±0.16^#^ | 0.32±0.15 | 0.24±0.11 | 0.3±0.08 |
|  | RT, μm | -3.7±8.24 | -7.1±4.73^#^ | -6±7.06 | -6.78±7.43^§^ | 0.39±7.51 |
|  | ChT, μm | -15.2±7.95^*^ | -14.8±7.61^#^ | -5±6.98 | -3.39±2.79 | 0.83±4.31 |

All data presented in Mean±SD, unpaired t-test applied. Normality and variance testing were conducted before the parametric test. Significant difference: one-way ANOVA multiple comparison *P* <0.05, *FDM vs Control2 ; # AAV-shCol4a2 vs Control2; † AAV-NC vs Control2; § Sham vs Control2;

| Forward ITR | 5’-GGAACCCCTAGTGATGGAGTT-3’ |
| --- | --- |
| Reverse ITR | 5’-CGGCCTCAGTGAGCGA-3’ |

Table S7. shRNA sequences

Table S8. Equipment and materials used in this study

| **REAGENT or RESOURCE** | **SOURCE** | **IDENTIFIER** |
| --- | --- | --- |
| **Instruments** | | |
| Keratometer | Nidek, Japan | ARK-30 |
| Streak retinoscope | 66Vision, China | YZ24 |
| A-Scan ultrasonography | SDK, China | SDK/180/ABS |
| SD-OCT | Heidelberg Engineering, Germany | SpectralisOCT |
| Fudus photography | TOPCON, Japan | TRC-NW8 |
| Mass spectrometer | Thermo Scientific, USA | Orbitrap Exploris^TM^ 480 |
| Confocal microscope | Carl Zeiss, Germany | ZEISS LSM 880 |
| **Primary Antibodies** | | |
| Collagen type IV Goat pAb | SouthernBiotech | Cat#1340-01 |
| Collagen type I Mouse mAb | Proteintech | Cat#67288-1-Ig |
| COL4A1 Rabbit pAb | Proteintech | Cat#30850-1-AP |
| COL4A2 Rabbit pAb | Proteintech | Cat#55131-1-AP |
| β-Actin Mouse mAb (8H10D10) | Cell Signalling Technology | Cat#12262 |
| Vinculin Mouse mAb | Proteintech | Cat#66305-2-Ig |
| **Secondary Antibodies** | | |
| Donkey anti-Goat IgG (H+L) Alexa Flour^TM^ 488 | Invitrogen | Cat#A11055 |
| Donkey anti-Goat IgG (H+L) Alexa Flour^TM^ 647 | Invitrogen | Cat#A32849 |
| Goat anti-Mouse IgG (H+L) Alexa Flour^TM^ 594 | Invitrogen | Cat#A-11005 |
| Donkey anti-Goat IgG (H+L) HRP | Invitrogen | Cat#A15999 |
| HRP-conjugated Donkey anti-Mouse IgG (H+L) | Proteintech | Cat#SA00001-8 |
| HRP-conjugated Goat anti-Rabbit IgG (H+L) | Proteintech | Cat#SA00001-2 |
| **Chemicals** | | |
| Proteinase Inhibitor | MCE | Cat#HY-K0010 |
| Phosphatase Inhibitor | MCE | Cat#HY-K0021 |
| RIPA Lysis Buffer | Beyotime | Cat#P0013B |
| 4’,6-diamidino-2-phenylindole (DAPI) | Sigma-Aldrich | Cat#D9542 |
| FAS fixing solution | Servicebio | Cat#G1109-50ML |
| oct compound | Beyotime | Cat#C0171A-118ml |
| **Commercial Eye Drops** |  |  |
| 1% cyclopentolate | Alcon, USA | CYCLOGYL® |
| 0.5%tropicamide/0.5%phenylephrine | XingQi, China | Zhuobian ® |
| 0.5%proparacaine hydrochloride | Alcon, USA | ALCAINE® |
| 0.3%tobramycin/0.1%dexamethasone | Alcon, USA | TobraDex® |
| **Critical Commercial Assays** | | |
| BCA protein assay kit | Beyotime | Cat#P0012 |
| Wes Seperation Module | ProteinSimple | Cat#SM-W002;  Cat#SM-W006 |
| Masson’s trichrome stain kit | Beijing Solarbio Science & Technology Co., Ltd., China | Cat#G1340 |
| Hematoxylin-Eosin stain kit | Beijing Solarbio Science & Technology Co., Ltd., China | Cat#G1120 |
| **Experimental Animals** | | |
| Pigmented rabbit | Danyang Changyi Breeding Co., Ltd |  |
| Pigmented guinea pig | Danyang Changyi Breeding Co., Ltd |  |
| **Oligonucleotides** | | |
| AAV-shCOL4A2 | Packgene Bio |  |
| AAV-NC | Packgene Bio |  |
| **Software and Algorithms** | | |
| Fiji-win64 | Schindelin et al.,2012. Schneider et al.,2012. |  |
| Proteome Discoverer | Thermo Scientific, USA | Version 2.4 |
| Compass for SW | ProteinSimple, USA | Version 6.3.0 |
| GraphPad Prism 9.5 for Windows 64-bit | GraphPad Software |  |
| SPSS Statistics 25 for Windows | IBM |  |
